# Optimizing myopia screening referral guidelines for children aged 4 to 18 based on non-cycloplegic indicators

**DOI:** 10.1186/s12886-025-04383-3

**Published:** 2025-10-10

**Authors:** Pingping Lyu, Jiaojiao Shi, Jingjing Wang, Xiangui He, Huijing Shi

**Affiliations:** 1 School of Public Health and Management, Jiangsu Vocational College of Medicine, 283 South Jiefang Road, Yancheng, 224005 China; 2 Department of Maternal, Child and Adolescent Health, School of Public Health, Fudan University, 138 Yixueyuan Road, Xuhui District, 200032 Shanghai, China; 3 Shanghai Eye Disease Prevention and Treatment Center, Shanghai Eye Hospital, Shanghai, China

**Keywords:** School vision screening, Referral criteria, Myopia prediction, Decision analysis

## Abstract

**Background:**

To evaluate the stability and predictive ability of uncorrected visual acuity (UCVA), non-cycloplegic refraction (NCR), and axial length (AL) as indicators in non-cycloplegic school vision screening for myopia.

**Methods:**

This retrospective cohort study is based on the Shanghai Child and Adolescent Large-scale Eye Study (SCALE). Participants included students who failed school screenings and were referred for follow-up cycloplegic refractions at eye hospitals within three months. We evaluated the differences in UCVA, spherical equivalent (SE), and AL between school screenings and hospital re-evaluations. Furthermore, we assessed the validity of using UCVA in combination with NCR as predictive metrics for myopia referral.

**Results:**

Among the 8,492 children, 4,357 (51.3%) were boys, with a mean age of 8.26 years (SD = 2.77). AL was identified as a reliable myopia screening indicator across all age groups (ICC = 0.981; 95% CI: 0.978–0.984), outperforming UCVA (ICC = 0.791) and SE (ICC = 0.806). The estimated prevalence of myopia using UCVA and NCR was 76.10%, significantly higher than 58.37% observed with cycloplegic testing. Sensitivity was 96.93% and specificity was 53.13% (Youden index = 0.5). In preschoolers, myopia rates decreased from 50.17% before to 19.82% after cycloplegia, while school-aged children exhibited better consistency. Decision curve analysis showed that the full model incorporating AL did not significantly benefit school-aged children, but may offer greater net benefits for preschoolers.

**Conclusions:**

AL should be integrated into screening programs for preschoolers. For school-aged children, the combination of UCVA and NCR suffices for myopia prediction, potentially eliminating the need for cycloplegia.

**Supplementary Information:**

The online version contains supplementary material available at 10.1186/s12886-025-04383-3.

## Introduction

Myopia has emerged as a major public health issue globally, particularly in East Asian countries [1–4]. China has one of the highest prevalence rates of myopia in the world, resulting in significant morbidity and an increasing trend [5–9]. The key to preventing and controlling myopia in children and adolescents lies in curbing its onset, slowing its progression, and managing complications.

The key to preventing and controlling the occurrence and progression of myopia in children and adolescents lies in inhibiting its onset, slowing its progression, and managing complications [10]. The school vision screening program can provide high-quality and cost-effective eye care services [11]. It not only monitors myopia risk factors and identifies early visual abnormalities in children, but also enables prompt communication with parents or guardians to refer children in need to specialized eye hospitals [12]. Consequently, the Chinese government has placed considerable emphasis on the role of school vision screenings and referrals in preventing and controlling myopia among children in recent years.

The American Association for Pediatric Ophthalmology and Strabismus (AAPOS) released guidelines in 2021, revising refractive thresholds to minimize unnecessary referrals while establishing new criteria for myopia referrals. A confirmatory ophthalmic examination includes a comprehensive cycloplegic assessment for children referred through vision screening or identified as high risk [13]. However, administering cycloplegic assessments to children in large-scale epidemiological studies, particularly with limited resources for cycloplegic refraction, can be challenging [14]. This issue is especially relevant in China’s school vision screenings, which involve nearly 300 million children. As a result, the National Health Commission of the People’s Republic of China has established specific referral criteria based on uncorrected visual acuity (UCVA) and non-cycloplegic refraction (NCR) to prevent and control myopia among all children and adolescents. However, evaluative research on the effectiveness of these criteria is lacking.

Setting referral criteria too low can lead to over-referral, thereby wasting time and medical resources [15], and can even cause adverse psychological effects on those referred [16]. Conversely, setting referral criteria too high can result in missed diagnoses, hindering early disease prevention and control. Appropriate referral indicator values should exhibit high repeatability and accuracy, reflecting the true visual health status of the screened population. Further research is needed to determine whether the myopia referral criteria in Chinese school vision screenings are appropriate.

Traditional evaluation metrics, such as sensitivity, specificity, area under the curve (AUC), and calibration, do not adequately inform clinical decisions. The thresholds justifying clinical implementation, particularly for sensitivity and AUC, remain ambiguous. Additionally, clinically acceptable calibration error is not well-defined, and there is no established framework for selecting between models that prioritize discrimination versus those that emphasize calibration. Decision curve analysis (DCA) is an innovative method for assessing the effectiveness of screening and diagnostic techniques. DCA evaluates diagnostic test performance in optimal settings while also considering real-world factors such as disease prevalence, treatment benefits, potential harms of misdiagnosis, and cost-effectiveness of the tests [17–20].

This study focuses on the application of various vision screening indicators from different schools in clinical practice, with an emphasis on clinical benefits and the comparison between different models. Therefore, decision curve analysis (DCA) is more suitable for this research. In summary, this study aims to investigate the appropriateness of current school vision screening referral criteria, providing a foundation and recommendations for further enhancing the referral standards for preschool and school-aged children in school vision screenings based on DCA.

## Methods

### Study design and data source

This is a diagnostic study based on the Shanghai Child and Adolescent Large-scale Eye Study (SCALE). Since 2012, kindergarten, primary, and junior high school students from all districts in Shanghai, China, have been gradually included for vision assessment. The baseline examination includes UCVA and NCR, with a subset of students also undergoing AL measurements. Students who fail the screening are referred to specialized eye hospitals for further examination and treatment. Referral decisions are primarily based on UCVA to determine the need for referral, while the degree of visual impairment and urgency for referral are assessed using NCR [21]. In the Shanghai Child and Adolescent Large-scale Eye Study (SCALE), the roles and responsibilities of participants are as follows: Teachers notify students and parents before screenings, providing essential information such as names, ages, genders, grades, and classes to community physicians. Community physicians then conduct vision screenings on school premises. After the screenings, they send results, a list of students needing further evaluation, and referral notifications back to the school. Teachers communicate these results to students and parents and issue referral notices as needed. Referred students, accompanied by their parents, visit designated hospitals for further ophthalmic examinations as indicated in their referral notices. Ophthalmologists document the follow-up outcomes based on these referrals and inform community hospitals, while community physicians input the follow-up data into the records.

This study focused on students from three geographically representative districts in Shanghai, including Jing’an, Minhang, and Pudong New Area, During the period from 2018 to 2020. Participants were identified through the SCALE after failing school-based vision screenings and were recommended for cycloplegic refraction examinations at eye hospitals within three months. By comparing the screening and follow-up results, we evaluated the repeatability and diagnostic capabilities of existing measures and proposed recommendations for myopia referral criteria for children aged 4 to 18 using Decision Curve Analysis (DCA).

The study involved three key steps. Step 1 assessed the consistency between school vision screening results and hospital reexaminations to investigate the repeatability of UCVA, NCR, and AL. Step 2 analyzed the concordance between myopia screening outcomes (UCVA combined with NCR) and cycloplegic refraction results to evaluate the predictive ability. Step 3 proposed recommendations for establishing myopia screening referral criteria based on findings from Steps 1 and 2.

### Study population

From 2018 to 2020, a total of 1,357,890 preschool and school-aged children in Jing’an, Minhang, and Pudong districts of Shanghai underwent vision screening. Among them, 623,061 students aged 4 to 18 years were referred Due to screening failure. However, only 46,268 of the referred students proceeded to hospitals for further diagnostic follow-up, and their results were recorded. Out of those, only 8,492 underwent cycloplegic refraction within three months as required to confirm myopia. This study focused on these 8,492 students. Our team previously analyzed the factors influencing the decision to refer students. The results indicated that factors such as student age, family economic status, eye care services provided by schools or community health centers, and students’ vision health significantly affect whether students seek hospital follow-up and utilize ophthalmic clinical services after vision screening [22]. 

**Fig. 1:**
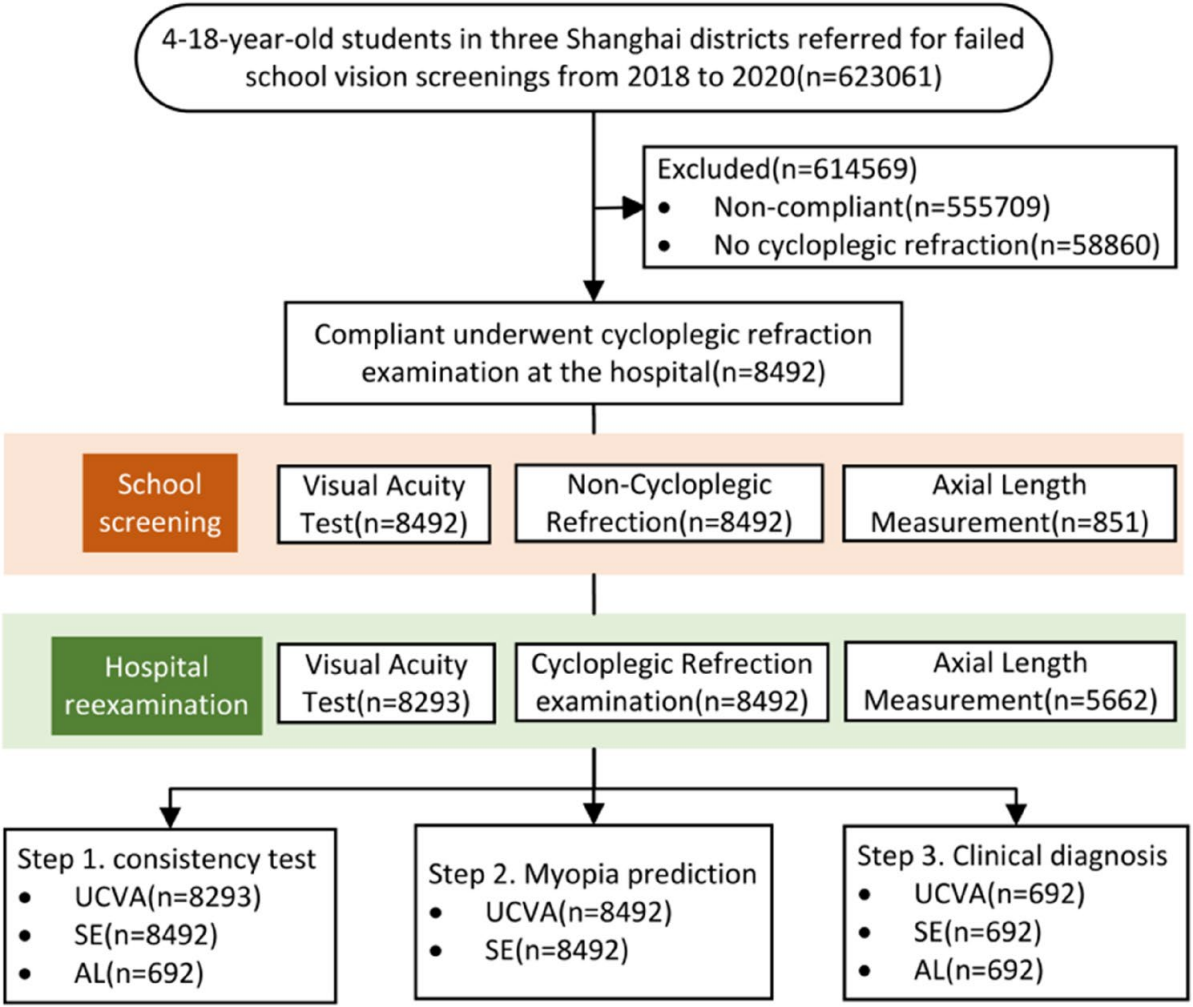
Flowchart for selection procedure of study participants

All 8,492 students underwent both UCVA and NCR examinations During the school vision screening, with 851 students receiving AL measurements. This study focused on students who had cycloplegic refraction data; however, not all of these students underwent visual acuity, non-cycloplegic refraction, and axial length measurements, resulting in inconsistent sample sizes for each metric. In the hospital reexaminations, all 8,492 students underwent non-cycloplegic refraction tests, 8,293 had UCVA checked, and 5,663 had their AL measured. In Step 1, the sample size for the UCVA consistency test was 8,293, while the sample size for consistency tests between NCR During screening and cycloplegic refraction in reexamination was 8,492, and for the AL consistency test, it was 692. In Step 2, the sample size for the myopia prediction test was 8,492, while in Step 3, the sample size for Decision Curve Analysis (DCA) was 692, as illustrated in Fig. 1.

### Analysis

Statistical analyses and figure creation were conducted using SAS 9.4 and R 4.3.2. Continuous variables were expressed as mean ± standard deviation (SD). Spherical equivalent (SE, SE = sphere + 0.5 × cylinder) derived from cycloplegic autorefraction was used as a gold standard for defining myopia, with a cutoff of SE ≤ −0.50 D. Screening myopia is defined as UCVA < 5.0 and NCR ≤ −0.50 D. The purpose of this Manuscript is to identify effective visual screening indicators for children throughout China, where the 5-point E-chart (GB/T 11533 − 2011 national standard) is exclusively used for routine vision screenings and documented in refractive development archives. All visual acuity data in this study were collected, analyzed, and presented using this standardized 5-point notation to reflect real-world clinical practice in China. The UCVA value of 5.0 is functionally equivalent to decimal 1.0 or Snellen 20/20, representing normal uncorrected visual acuity. Only data for the right eye were analyzed due to the high correlation between the two eyes [23]. 

The use of Bland-Altman plots is advantageous in situations involving repeated measurements and calibrations [24]. Bland-Altman plots involve plotting the differences between two methods against the mean of the two values and then using these data to derive the limits of agreement (LOA), within which 95% of the differences are expected to fall. The LOA are calculated as the mean difference ± 2 SD [25]. At the group level, the majority of values fell within the LOA (2 SD), indicating a fairly good level of agreement [26]. Further, the intraclass correlation coefficient (ICC) [27] can be utilized to demonstrate the consistency of measurement outcomes and identify systematic differences. Simultaneously calculating both the percent agreement and kappa value can effectively evaluate the extent to which the collected data accurately reflect the variables being measured [28]. 

The decision curve is based on a decision-theoretical framework that takes into account both the benefits and costs of interventions to patients [29]. The Decision Curve Analysis (DCA) calculated the net benefit for each model at threshold probabilities from 1 to 99%, balancing true positive gains against false positive harms using threshold-dependent weighting. Two benchmark strategies were assessed concurrently: the “Treat None” approach (net benefit = 0), which indicates no interventions, and the “Treat All” strategy, which derives net benefit from population prevalence. Decision curves were generated by plotting model-specific net benefits against threshold probabilities, with uncertainty determined through 1,000 bootstrap iterations to establish 95% confidence intervals. The focus was on identifying threshold ranges where a model’s net benefit exceeded both benchmarks, indicating clinical applicability. Comparisons distinguished between uniform dominance (consistent superiority across all thresholds) and threshold-specific advantages (superiority within certain probability ranges). Finally, clinical impact curves (CIC) were plotted to evaluate the clinical usefulness and applicability of the net benefits of the model with the best diagnostic value [30]. A *p*-value < 0.05 was considered statistically significant.

In Step 1, this study assessed the repeatability and stability of using UCVA, NCR, and AL as screening indicators in school vision programs by constructing Bland-Altman plots and calculating ICC. Step 2 involved evaluating the predictive ability of combining UCVA and NCR for myopia by analyzing percent agreement, kappa values, sensitivity, specificity, and the Youden index. Finally, Step 3 employed decision curve analysis (DCA) to offer insights and recommendations for the referral decisions of myopia screening practitioners for students.

## Results

### Baseline characteristics

Among the 8,492 children, 4,357 (51.3%) were boys, with a mean age of 8.26 years (SD = 2.77). All participants underwent UCVA and NCR examinations During school vision screening, with 851 students receiving AL measurements. The mean values for UCVA, NCR, and AL were 4.71 (SD = 0.26), −1.27 (SD = 1.63), and 23.44 (SD = 1.27), respectively. Based on educational stage, preschool children (28.0%) exhibited superior performance in UCVA, NCR, and AL compared to school-aged children. Additionally, the differences in SE before and after cycloplegia (0.90 D) were more pronounced in preschool children than in school-aged children (0.27 D), as demonstrated in Table 1. Additionally, the prevalence of isometropia (spherical equivalent difference ≤ 0.75 D) in our study was 76.6% (6,501/8,492), while anisometropia (interocular difference ≥ 1.00 D) affected 19.7% of subjects (1,673/8,492).

**Table 1:** Study population characteristics by educational stage

Variable	Total	Pre-school	School-age
Participants, n(%)	8492	2376(28.0)	6116(72.0)
Age(y), mean±SD	8.26 ± 2.77	4.99 ± 0.85	9.55 ± 2.15
Sex(%boy)	4357(51.3)	1278(53.8)	3079(50.3)
UCVA (5-grade notation), mean±SD	4.71 ± 0.26	4.81 ± 0.14	4.67 ± 0.28
Precycloplegia SE (D), mean±SD	-1.27 ± 1.63	-0.41 ± 1.35	-1.61 ± 1.61
Postcycloplegia SE (D), mean±SD	-0.83 ± 1.84	0.49 ± 1.40	-1.34 ± 1.67
Axial length (mm), mean±SD	23.44 ± 1.27	22.48 ± 0.80	24.13 ± 1.09

*Abbreviations UCVA* uncorrected visual acuity, *SE* spherical equivalent, *D* diopters, *AL* axial length

### Agreement assessment of myopia screening indicators

**Fig. 2:**
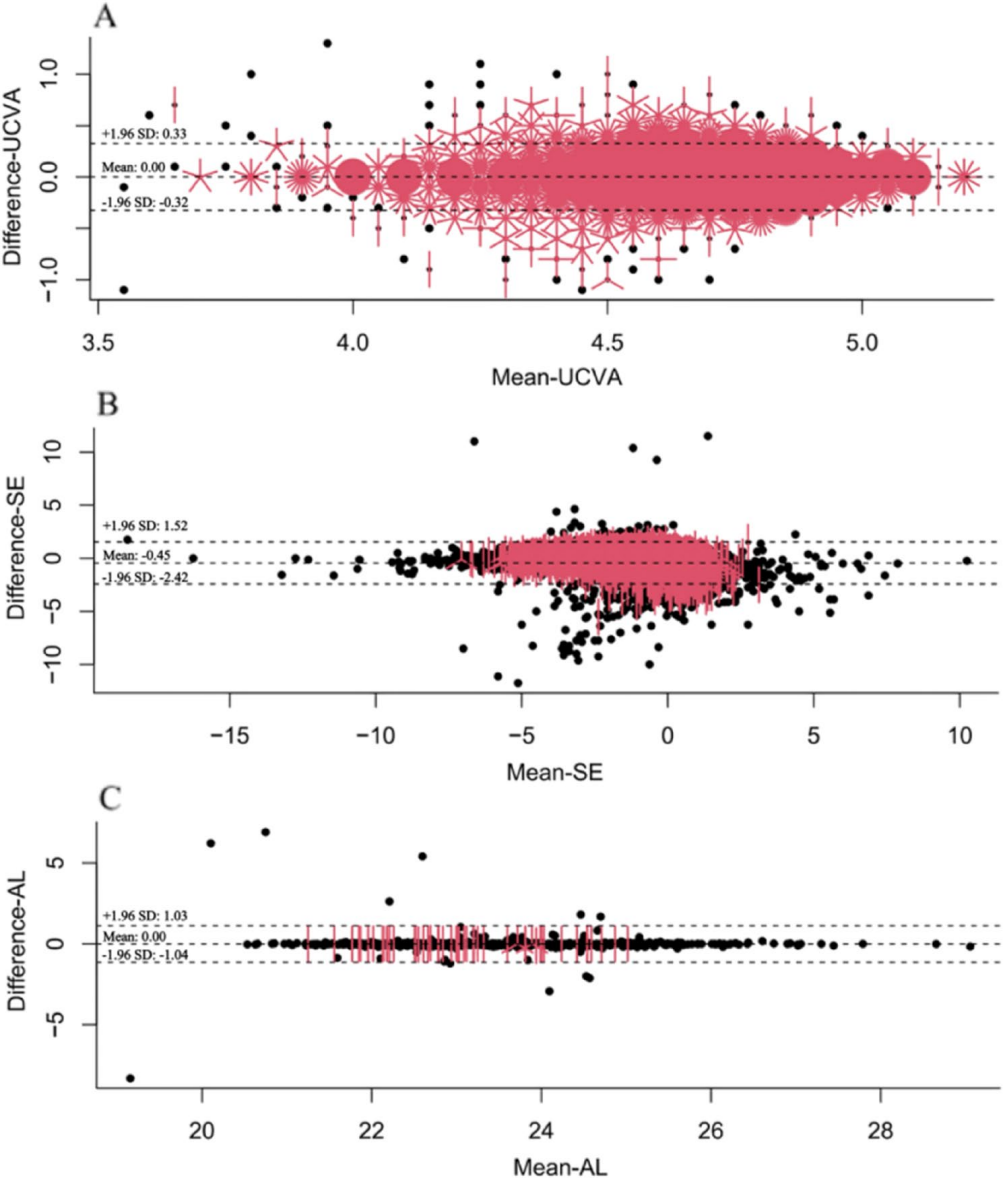
Bland-Altman plot comparing the agreement between uncorrected visual acuity (UCVA) at school screening and hospital re-examination (Figure **A**) and axial length (AL) (Figure**C**) as well as the pre-cycloplegia spherical equivalent (SE) at school screening and post-cycloplegia SE at hospital re-examination (Figure**B**). The central line represents the absolute average difference, while the upper and the lower lines represent±1.96 standard deviation. In Figure **B**, the units of the axes are all in diopters (D), while in Figure **C**, the units of the axes are all in millimeters (mm)

Bland-Altman plots in Fig. 2 illustrate the discrepancies in the school vision screening and hospital re-examination results for each participant. At the group level, the majority of AL values fell within the limits of agreement (LOA) (2 SD), indicating a substantial level of agreement. Combined with the ICC, this demonstrates that the repeatability, reliability, and stability of AL as a myopia screening indicator (ICC = 0.981, 95% CI: 0.978, 0.984) were significantly superior to those of UCVA (ICC = 0.791, 95% CI: 0.782, 0.799) and SE (ICC = 0.806, 95% CI: 0.710, 0.862).

**Fig. 3:**
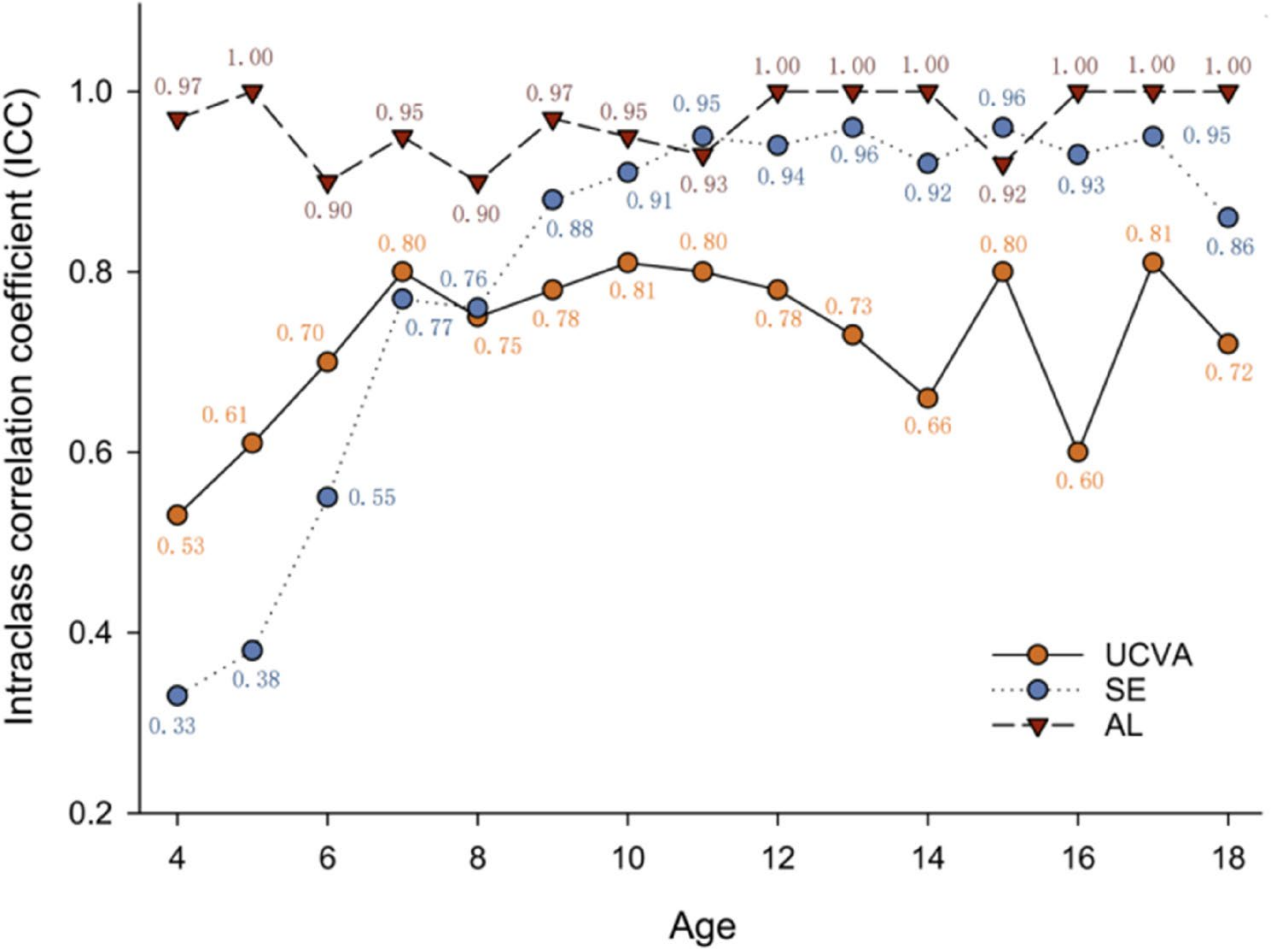
Changes in Intraclass Correlation Coefficient (ICC) in Children Aged 4 to 18 Between Uncorrected Visual Acuity (UCVA) at School Screening and Hospital Re-examination, as well as Axial Length (AL) and Spherical Equivalent (SE) Measurements Pre- and Post-Cycloplegia. In Figure 3, the unit of the x-axis is years, the y-axis starts at 0.2 with an interval of 0.2

Figure 3 illustrates the age-related variations in the consistency of the UCVA, SE, and AL indices between screening and re-examination. The AL Maintained a high level of consistency throughout the 4-18 age range, with ICC values consistently exceeding 0.9. For preschool children aged 4-6, both UCVA and SE exhibited relatively low consistency at age 4, with ICC values of 0.53 and 0.33, respectively. The consistency of these two indices increased with age. In the schooling phase for ages 7-18 years, although SE initially had lower values than UCVA, after 7-8 years, the consistency of SE gradually surpassed that of UCVA, approaching the level of AL, while the consistency of UCVA notably lagged behind AL and SE after 7-8 years.

The consistency of SE was assessed by comparing non-cycloplegic refraction results at screening with cycloplegic refraction results at re-examination. The repeatability of SE was notably lower than that of AL, suggesting significant differences in refraction results Due to cycloplegia for children aged 4-6. However, for school-aged children, the consistency of SE gradually improved before and after cycloplegia, becoming able to substitute for AL after the age of 8. While there was a moderate enhancement in the consistency of UCVA during the preschool stage, its long-term consistency remained relatively low.

### Myopia predictive capability of the combined use of UCVA and NCR tests

Tables 2 and 3 reveal that out of 4,957 myopic children diagnosed using the gold standard, 152 (3.07%) remained undiagnosed with myopia when using the combined tests. Among the 3,535 non-myopic children identified by the gold standard, 1,657 (46.87%) were incorrectly diagnosed with myopia using the combined tests. The Youden index was 0.50, with an agreement rate of 0.79 and a kappa value of 0.53 .

**Table 2:** Accuracy of the combination of UCVA and NCR tests for myopia screening by educational stage

Group	UCVA and NCR tests	Cycloplegic Autorefraction Test		
		Positive	Negative	Total
Total	Positive	4805	1657	6462
	Negative	152	1878	2030
	Total	4957	3535	8492
Preschool	Positive	408	787	1195
	Negative	63	1118	1181
	Total	471	1905	2376
School-aged	Positive	4397	870	5267
	Negative	89	760	849
	Total	4486	1630	6116

**Table 3:** Reliability of the combination of UCVA and NCR for myopia screening by educational stage

Group	% Agreement	Kappa (95%CI)	Sensitivity(%)	Specificity(%)	Yonden’ Index
Total	0.79	0.53(0.50,0.55)*	96.93	53.13	0.50
preschool	0.64	0.29(0.26,0.32)*	86.62	58.69	0.45
school-aged	0.84	0.53(0.50,0.55)*	98.02	46.63	0.45

*p＜0.05

The sensitivity of the combined tests was 96.93%, while the specificity was 53.13%. The false negative rate was 3.07%, indicating that the tests demonstrated a strong ability to identify myopia, as the vast Majority of actually myopic children received accurate diagnoses. Conversely, the false positive rate was 46.87%, which suggests that the tests were less effective at excluding non-myopic children, resulting in a significant proportion of non-myopic children being incorrectly classified as myopic. Therefore, there is a need to further optimize the testing methods to reduce this misdiagnosis. Compared to school-age children, preschoolers exhibited lower levels of agreement, kappa values, and sensitivity. The agreement rate among preschoolers was only 0.64, with a kappa value of 0.29.

### Selection of myopia screening indicators/referral criteria through decision curve analysis

**Fig. 4:**
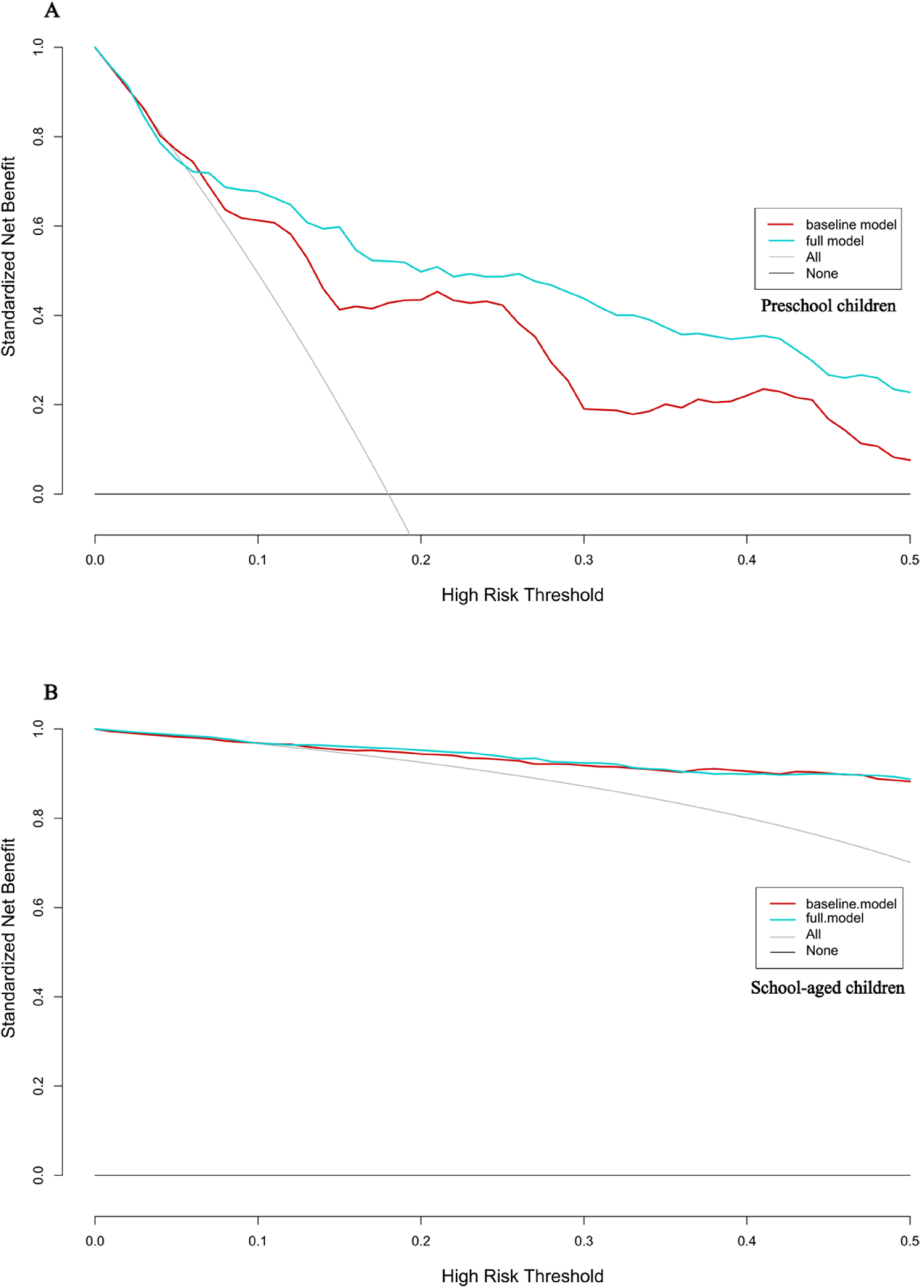
Decision curves for the two multivariate myopia prediction models

Figure 4 presents the decision curve analysis (DCA), indicating that for preschool children, the full model including age, sex, UCVA, NCR, and AL significantly outperforms the baseline model (UCVA + NCR) in terms of net benefit. However, for school-aged children, there is no significant difference in net benefit between the two myopia prediction models.

The vertical axis represents the net benefit value, while the horizontal axis indicates the threshold level (i.e., possible probability cut-points). The decision curve is generated by plotting the net benefit as a function of threshold levels. Net benefit is defined as the benefit derived from the prediction model minus the loss incurred due to incorrect predictions. The blue line represents the baseline model, which includes UCVA combined with NCR, while the red line represents the full model that includes age, sex, UCVA, NCR, and axial length (AL). The “none” model signifies the “treat none” approach, where no treatment or intervention is provided to any subjects, regardless of the threshold (i.e., all subjects are considered negative). Conversely, in the “treat all” model, treatment or intervention is applied to all subjects, treating everyone as positive, irrespective of the threshold. Figure A illustrates the decision curve analysis (DCA) for preschool children, while Figure B depicts the DCA for school-aged children.

**Fig. 5:**
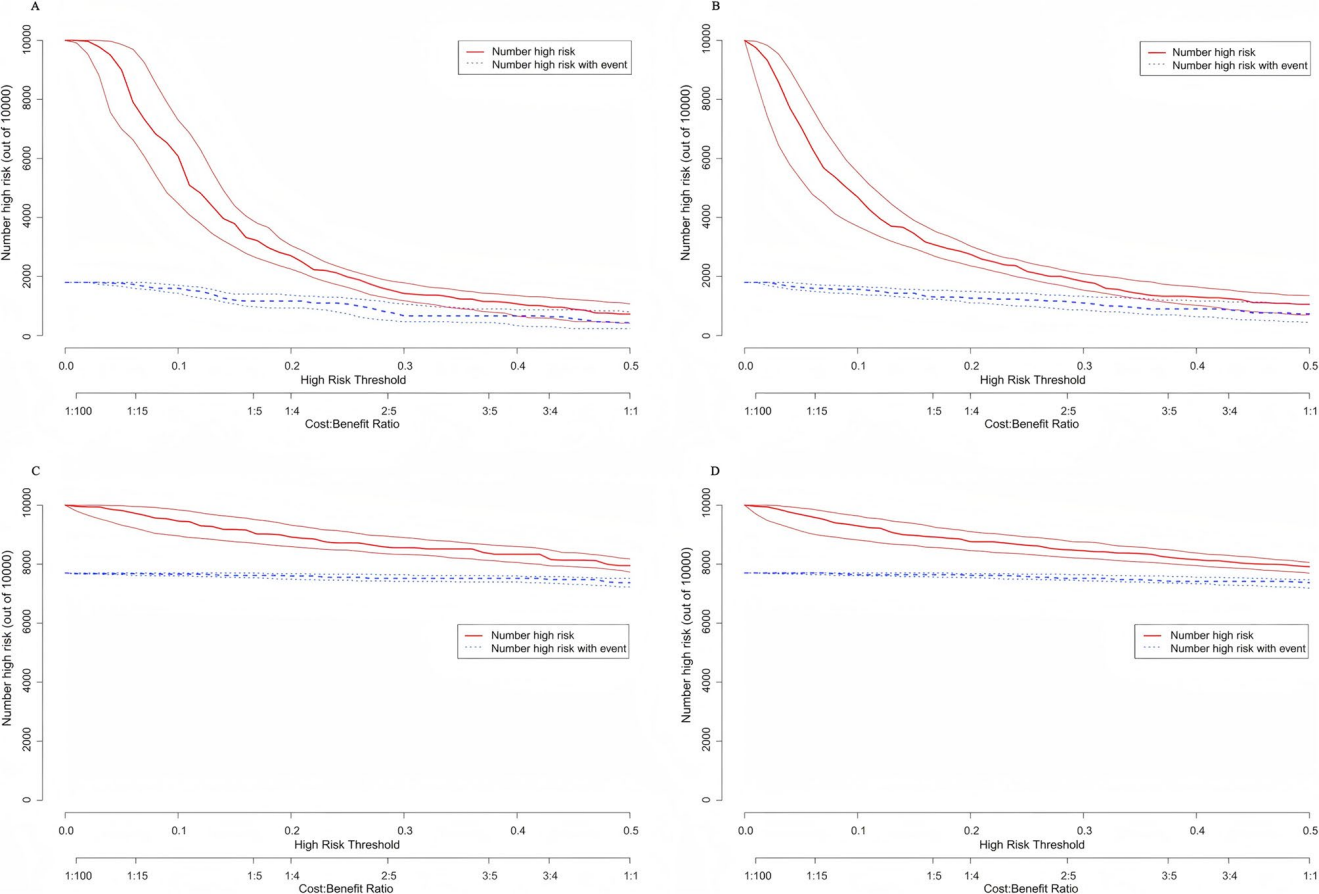
Clinical impact curve (CIC) for the two multivariate myopia prediction models. The red curve represents the number of individuals classified as high risk (positive) by the model at each threshold probability, while the blue curve represents the number of true positives at each threshold probability. The two x-axes represent the high risk threshold and cost benefit ratio. The high risk threshold indicates the boundary at which study subjects are considered to have a higher risk. When this threshold is exceeded, further examinations, interventions, or preventive measures are typically recommended for patients. The cost benefit ratio is a metric used to assess economic efficiency, calculated by comparing the costs of an investment or intervention to the net gains obtained. **A** displays the baseline model for preschool children, **B** shows the full model for preschool children, **C** depicts the baseline model for school-aged children, and **D** illustrates the full model for school-aged children

The clinical impact curve (CIC) analysis presented in Fig. 5 evaluates the clinical applicability of the myopia risk prediction nomogram. Overall, both myopia prediction models overestimated the prevalence of myopia, particularly the baseline model for preschool children (Figure A), which exhibited the highest false positive rate within a threshold range of 0 to 0.3. In contrast, both models demonstrated good predictive performance for school-aged children. The results of the k-fold cross-validation for both models, demonstrating the stability and reliability of the models, can be found in Supplementary Material eFigure 1.

## Discussion

This study evaluates current myopia screening strategies in China, which primarily rely on UCVA and NCR for prediction and referral. Our findings indicate that, for preschool children, the combination of UCVA and NCR does not accurately predict myopia, resulting in a high rate of false positives. In contrast, AL is a more reliable metric for predicting myopia and determining referrals, especially when combined with age and gender. School-aged children show strong consistency in SE before and after cycloplegia, suggesting that a more complex predictive model May not provide significant benefits The study included 8,492 participants, with 72.0% being school-aged and 28.0% preschool children. Compared to other studies [31–34], the UCVA and NCR levels of participants in this research were relatively poor, as the subjects were those specifically referred for further examination due to initial screening failures. The current indicators (UCVA combined with NCR) overestimate the prevalence of myopia, reporting 76.10% compared to 58.37% with cycloplegic testing. The sensitivity was 96.93%, but the specificity was only 53.13%. Non-cycloplegic refraction may fail to neutralize ciliary muscle accommodation, significantly overestimating myopia prevalence. Consequently, many non-myopic children are misclassified as myopic, reducing specificity. This is well-documented in the literature. For example, Barry and König examined 27 three-year-old children without cycloplegia and found that non-cycloplegic screening produced numerous false-positive referrals with low specificity (0.58) [35]. Similarly, Choong et al. tested 117 schoolchildren with and without cycloplegia, demonstrating that all non-cycloplegic autorefractors overestimated myopia [36]. The prevalence of myopia in preschool children was 50.17% before cycloplegia, decreasing to 19.82% afterward. The difference in SE measurements before and after cycloplegia was significantly greater in preschool children (0.90 D) than in school-aged children (0.27 D), mirroring findings from previous research [37, 38]. Although some studies support the effectiveness of cycloplegic refraction for predicting myopia, they also recognize the value of AL measurements [39–42]. Given the difficulties in conducting cycloplegic exams for all children during school screenings, AL serves as a valuable alternative Our study found that repeatability of AL measurements (ICC = 0.981) was significantly higher than that of UCVA (ICC = 0.791) and SE (ICC = 0.806), especially among preschool children. For 4-year-olds, SE consistency was low (0.33) compared to UCVA (0.53) and AL (0.97). The low consistency of UCVA may stem from the universal use of the E-chart as the testing chart in China, regardless of age. While various conversion formulas exist [23], some studies suggest that Lea symbols are more effective for younger children than the E-chart [43]. Xinxin Yu et al. [44] also reported that the repeatability of axial length (AL) measurements in children is relatively high Numerous studies [45–49] have indicated that vision levels vary among children of different ages and sexes. However, current myopia prediction and referral criteria do not account for these two factors. Therefore, this study also included age and sex in the full model. We compared the baseline model (UCVA combined with NCR) with a full model that incorporates additional factors such as gender, age, and AL. Comparative results reveal that using the full model as a predictive tool for school-aged children does not offer significant advantages, as the combination of UCVA and NCR already achieves satisfactory predictive accuracy, consistent with other studies [20, 23, 50]. However, for preschool children, employing the full model as a standard for predicting myopia and determining referrals yields a higher net benefit. In preschool vision screening programs, the cost for each confirmed case can reach several hundred dollars [51]. Thus, this study advocates for the inclusion of AL in the examination protocols for preschool children to reduce false positive rates and to use the simplified combination of UCVA and NCR as predictors and referral criteria for myopia in school-aged children to minimize the waste of medical resources.

### Limitations

This study has several limitations. The exact number of children who consulted an ophthalmologist without reporting back to the screening organization is unknown, creating uncertainty in cases where no response is received. However, the data collected encompass all designated follow-up hospitals, and teachers often guide parents to seek re-examination at these facilities. During the follow-ups, some students were excluded from the analysis due to missing cycloplegic refraction data; only students with complete data were included. The absence of cycloplegic refraction may result from physicians determining that a cycloplegic examination is unnecessary based on the uncorrected visual acuity, suggesting a lower likelihood of myopia. This further supports the study’s assertion that the combination of uncorrected visual acuity and non-cycloplegic refraction increases the risk of false positives. Moreover, this study did not consider the economic factors associated with various myopia indicators. Although the prices of axial length measurement devices have significantly decreased in recent years, they remain unaffordable in many rural and underdeveloped areas. This may be the greatest barrier to utilizing axial length for school vision screenings.

## Conclusion

AL measurements should be incorporated into school vision screening programs for preschool children. A more comprehensive myopia referral standard should be developed based on multiple indicators, including age, sex, UCVA, NCR, and AL, along with the clinician’s experience. For school-aged children, the combination of UCVA and NCR is sufficient to achieve satisfactory myopia prediction results, while potentially eliminating the need for cycloplegia.

## Supplementary Information

Supplementary Material 1.

## Data Availability

The datasets generated and analyzed during the current study are available. The materials used in this study are available. Requests for access to the data and materials should be directed to the corresponding author (Huijing Shi, hjshi@fudan.edu.cn).
