# Supplementary figures and images for: Optimizing myopia screening referral guidelines for children aged 4 to 18 based on non-cycloplegic indicators
Source: BMC Ophthalmol. 2025 Oct 10;25:561. doi: 10.1186/s12886-025-04383-3 (PMC12512548; doi:10.1186/s12886-025-04383-3)

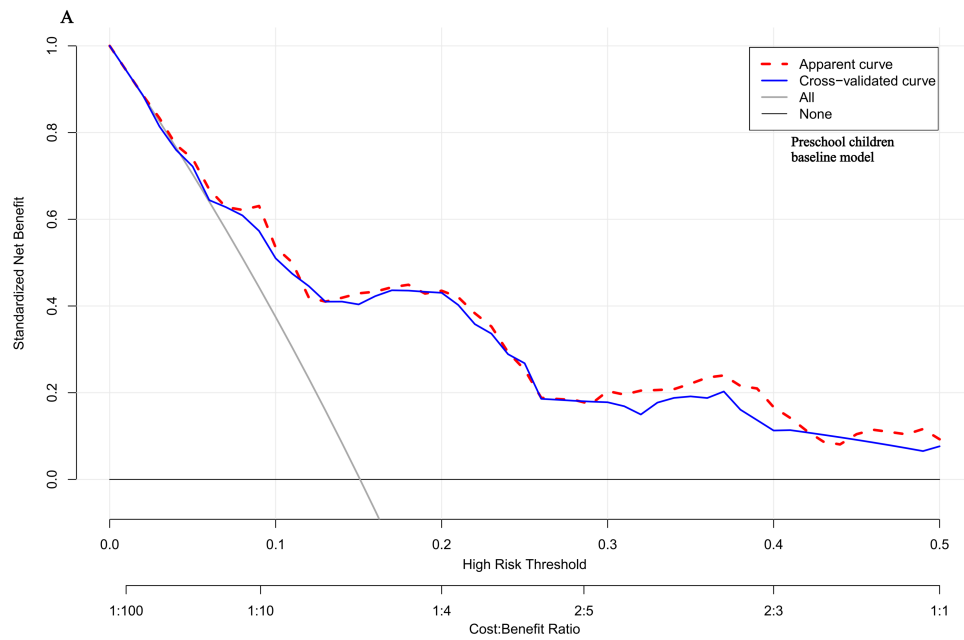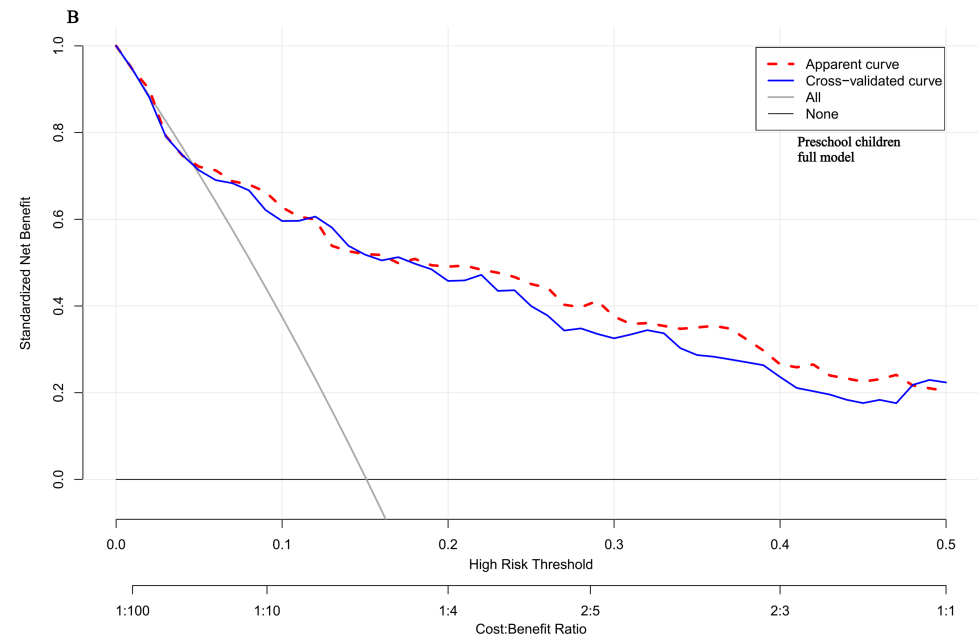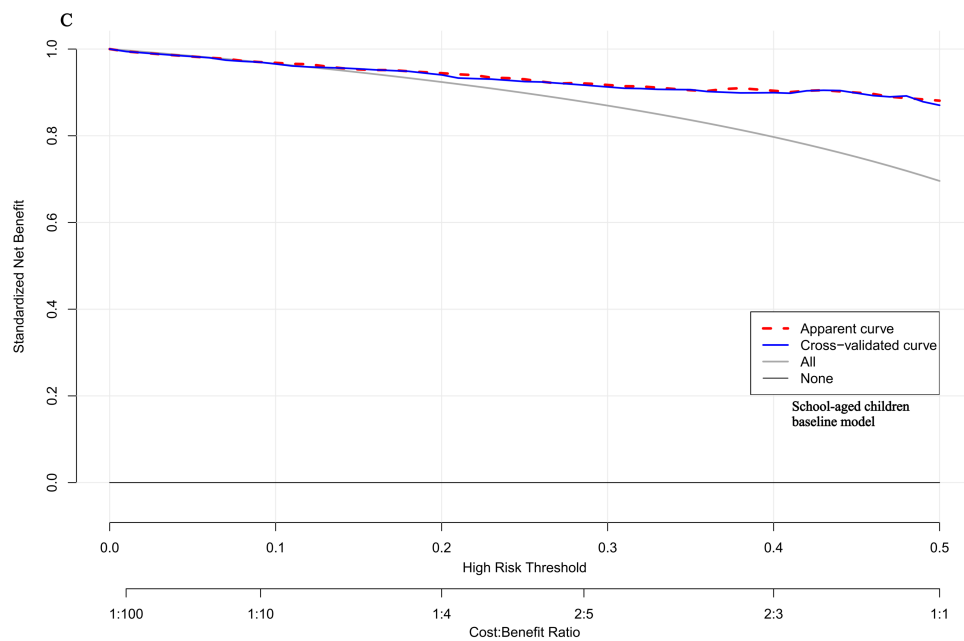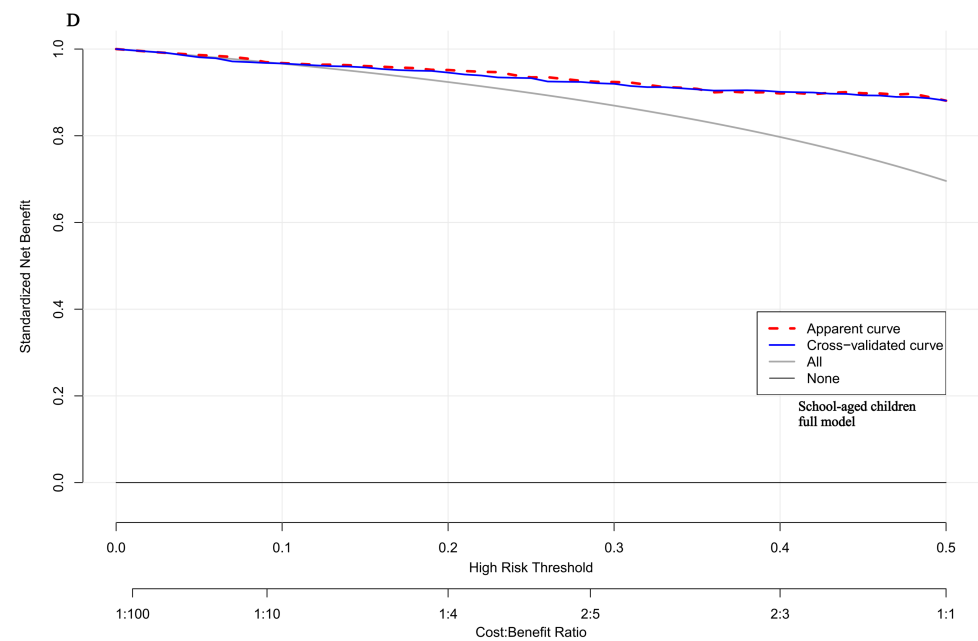

Supplement: Supplementary file 1 — Supplementary Material 1. [file 12886_2025_4383_MOESM1_ESM.pdf]
